# Supplementary material for: An epithelial signalling centre in sharks supports homology of tooth morphogenesis in vertebrates
Source: eLife. 2022 May 10;11:e73173. doi: 10.7554/eLife.73173 (PMC9249395; doi:10.7554/eLife.73173)
Supplement: Supplementary file 2. — Primer sequences used to generate the RNA probes for in situ hybridisation in Figures 2—5 (and Figure 2—figure supplement 1). [file elife-73173-supp2.docx]

| Gene | Forward Primer | Reverse Primer |
| --- | --- | --- |
| *lef1* | CATGCACTCTACAGGGATCCC | TCTGGATCAGAGTCTTGCTGC |
| *β-catenin* | AGTGGTTAAGCTACTGCACCC | AAGCTAGCATCATCTGGACGG |
| *shh* | TGACTCCCAATTACAACCCGG | TCAGGTCCTTCACTGACTTGC |
| *mdk* | GACAGGGTCCTCTGAAGCTG | TTAGGGTTCCATTGCGAGTC |
| *fgf3* | CTTGTTGCTGAGTCTTCTGGC | AACTCTTCAGCAGGTTCTCCC |
| *fgf10* | TGGATACTGACAAAGGGTGCC | GACATCGTGTCTCACCACTATTGG |
| *wnt11* | TCTGACATGAGGTGGAACTGC | TCTCTTGAGTTCCGTTGGAGC |
| *dkk1* | TGCCTCTACAATGTCGTGAGC | GTGCAGCCTCGAATTCTTGC |
| *bmp4* | GGAGCACAGGTCTATGGAAAGG | GGAGCACAGGTCTATGGAAAGG |
| *smad1* | GGAATCCGAGACACTCTTGGC | TTCAACAACCAGCTCTTCGCG |
| *smad3* | TAGTCACCATGAGCTTCGAGC | CCAATGTGCCTTCTTGTCAGC |
| *isl1* | ATTGTTCGGGACTAAATGCGC | TGCAGCGTTTGTTCTGAAACC |
| *jag1* | GGGCGACACTATAGAGAAGGC | ACAGGGATCAGAGATGCAAGC |
| *jag2* | AGCTGTACTGTGGCAATCTCC | GGATGCAACTGCTGTTCTTCG |
| *bambi* | GCATCTAACTGTGTGGCAACG | TCCAAGTCTAACTTCGCCACC |
| *sfrp3* | CCGTCATGAGGAGGTACAACC | TTCTGTTCCTCTGCTTCGACG |
| *smad7* | TCCTTGCCGGTACTGATATGC | GTGTGAAATCGTGGTCGTTGG |
| *runx2* | ATCTCTCAATCCTGCACCAGC | CCAGACAGACTCATCAATCCTCC |
| *wwtr1* | AGTTCCAGGTTCAGCACATCC | GCATTAGGTCCTCGCCTTCC |
| *foxl1* | TCAGAGGGTGACATTGAACGG | CTGATGGAGAAGGGTTGGACC |
| *foxl1l* | GTATCTCGACCTGCCTACAGC | ATGTCAGATGCCCAGTCTTGG |
| *notch2* | AGAATGGAGGCACTTGTCAGG | AGCCTCCTTTGTTCAGACAGG |
| *msx2* | TCACCGAAGTATTGTGCCTCC | GGAGCACAGGTCTATGGAAAGG |
| *axin2* | GACGGACAGTAGCGTAGATGG | TGGTGGATGTGATGATGGTGG |
